# Heart Failure With Mid-range Ejection Fraction: Every Coin Has Two Sides

**DOI:** 10.3389/fcvm.2021.683418

**Published:** 2021-07-21

**Authors:** Kaiyuan Zhu, Teng Ma, Yang Su, Xin Pan, Rongrong Huang, Fenglei Zhang, Chunxi Yan, Dachun Xu

**Affiliations:** ^1^Department of Cardiology, Qidong People's Hospital, Nantong, China; ^2^Department of Cardiology, Shanghai Tenth People's Hospital, Tongji University School of Medicine, Shanghai, China; ^3^Department of Geriatrics, Shanghai Tenth People's Hospital, Tongji University School of Medicine, Shanghai, China

**Keywords:** heart failure, heart failure with reduced ejection fraction, heart failure with mid-range ejection fraction, heart failure with preserved ejection fraction, left ventricular ejecting fraction

## Abstract

This review summarizes current knowledge regarding clinical epidemiology, pathophysiology, and prognosis for patients with HFmrEF in comparison to HFrEF and HFpEF. Although recommended treatments currently focus on aggressive management of comorbidities, we summarize potentially beneficial therapies that can delay the process of heart failure by blocking the pathophysiology mechanism. More studies are needed to further characterize HFmrEF and identify effective management strategies that can reduce cardiovascular morbidity and mortality of patients with HFmrEF.

In 2013, the American College of Cardiology/American Heart Association defined “Heart failure with borderline ejection fraction” as heart failure with typical clinical symptoms and LVEF of 41–49%. In 2016, the European Society of Cardiology (ESC) firstly classified heart failure into three categories based on the LVEF: heart failure with reduced ejection fraction (HFrEF, LVEF<40%), heart failure with mid-range ejection fraction (HFmrEF), and heart failure with preserved ejection fraction (HFpEF, LVEF≥50%). The LVEF range of HFmrEF is 40~49%. The new classification encourages further researches on HFmrEF, as it reflects a median phenotype between HErEF and HFpEF, and the subtype of heart failure may correspond to different stages during the development, which is inconsistent with the results of current clinical studies. Does HFmrEF represent an independent type or a transitional stage between HFrEF and HFpEF? Do the targeted therapies known to be efficacious for HFrEF patients have beneficial effects on patients with HFmrEF? This article summarizes the current understanding of the HFmrEF and discusses how to better manage patients with HFmrEF.

## Definition and Diagnosis

Modern treatment of HF is primarily dependent on the objective evaluation of LVEF, which can predict adverse outcomes even in the absence of symptoms. In the past, patients with heart failure (HF) had been categorized into heart failure with reduced ejection fraction (HFrEF, LVEF<40%) and heart failure with preserved ejection fraction (HFpEF, LVEF≥50%), while patients with an LVEF value in the range of 40–49% were considered in the “gray area.” In 2016, ESC defined patients with LVEF in the range of 40–49% as HF with mid-range ejection fraction (HFmrEF) in order to stimulate researches on the underlying characteristics, pathophysiology, and treatment of this subtype of patients (1).

For a precise diagnosis and treatment, the introduction of this new classification is understandable and reasonable. 2017 ACC/AHA guidelines for the management of heart failure and Chinese guidelines for the diagnosis and treatment of heart failure in 2018 adopt the same definition (2, 3). And the need for identifying this new subgroup has made HFmrEF a new research hotspot.

Although HFmrEF was first introduced into the guidelines in 2016, the “gray area” between HFrEF and HFpEF had already been mentioned in 2012 ESC guidelines (4). Therefore, the guidelines merely legitimized this “gray area” as a distinct entity by giving it a name (5). The primary purpose for defining this new group is to highlight its importance and stimulate researches relevant to these patients populations, as they are typically excluded from both HFpEF and HFrEF trials. As a result, it also confused many physician, including clinical presentation, management, and outcomes of HFmrEF, which partially overlaps with HFrEF and HFpEF. OPTIMIZE-HF and ADHERE studies have begun to explore the characteristics, treatment patterns, and clinical outcomes of patients with a mild decrease in LVEF, finding that these patients may be significantly different from HFrEF and HFpEF populations (6, 7).

## Epidemiological Characteristics

### Prevalence

More than 6.5 million people have been diagnosed with HF in the United States (8). Relevant research has shown that HFmrEF accounts for 13–24% of HF, meaning that there are about 1.6 million HFmrEF patients in the United States (5, 9). From 2005 to 2010, the proportion of HFpEF patients increased from 33 to 39%, the proportion of HFrEF patients decreased from 52 to 47%, and the proportion of HFmrEF patients was relatively stable (increased from 13 to 15%) (10). The PINNACLE registration study, the largest descriptive analysis of HFmrEF patients to date, determined that 36.1% of all is HFrEF patient, 7.5% is HFmrEF, and 56.5% is HFpEF (11). Continuous hospitalization data from a multicenter ADHF in Japan showed that 651 (17.1%) of 3,572 patients were categorized with HFmrEF. Of 3,580 patients with heart failure in a recent Spanish report, HFmrEF patients were found in 14% (12). The unimodal distribution of LVEF deciles shows that a large number of patients fall within the “middle zone” of LVEF; the prevalence rate of this medium-range is estimated to be 10–20%, as most patients have no clinical symptoms of heart failure according to CHARM reports (13).

### Readmission Rate and Mortality

The readmission rate of HFmrEF is between those of HFrEF and HFpEF. In the GWTG-HF registry, all-cause readmission rates of HFmrEF patients were 20.9 and 63.2% within 30 days and 1 year, respectively. These numbers are similar to those of HFpEF patients (20.5 and 62.5%, respectively) and slightly higher than those of HFrEF patients (19.7 and 59.6%, respectively). However, the readmission rates for cardiovascular events in patients with HFmrEF (11.3% within 30 days, 41.6% within 1 year) were higher than those of the HFpEF group (9.9 and 37.4%, respectively) and close to those of the HFrEF group (12.9 and 42.4%, respectively) (14). Compared to HFrEF and HFpEF patients, the specific HF readmission rate for HFmrEF patients was intermediate. The GUIDE-IT trial used NT-pro-BNP to guide the treatment of patients with HFrEF; however, the study was terminated prematurely due to inefficacy (15).

Of all HF patients, the HFrEF group had the highest mortality, and the mortality of the HFmrEF group was similar to that of the HFpEF group. In the OPTIMIZE-HF study, the overall in-hospital mortality rate of HFrEF patients was 3.9%, in comparison to 3.0% for HFmrEF patients and 2.9% for HFpEF patients (6). A Canadian study of HF inpatients showed that the untreated mortality rate of HFmrEF patients was 5.1% within 30 days and 21.3% within 1 year, which were intermediate compared to those of HFpEF patients (5.3 and 22.2%, respectively) and HFrEF patients (7.1 and 25.5%, respectively), but the differences were not statistically significant (16). A meta-analysis of individual data from nearly 40,000 HF patients showed that, for patients with LVEF<40%, mortality increased gradually with every 5–10% decrease in LVEF, but there was no significant difference in LVEF>40% group (17).

A recent study by the Swedish Heart Failure Registry (Swede-HF) found that chronic kidney disease in patients with HFmrEF and HFrEF was a stronger predictor of mortality than HFpEF (18). In another study, HFmrEF patients over the age of 85 and those with the chronic obstructive pulmonary disease had a higher risk of death within 1 year after discharge than similar patients in other HF groups. Based on GWTG-HF registrations from 2005 to 2010, although the unadjusted hospital mortality rate of HFpEF patients decreased from 3.32 to 2.35%, the mortality rates of HFmrEF patients (2.69–2.88%) and HFrEF patients (3.03–2.83%) were relatively stable (10). Within each HF group, physiological factors, and concurrent disease contribute to 1-year mortality rates to varying degrees. Age over 85 years old and co-occurrence of COPD were more strongly correlated with 1-year mortality in HFmrEF patients (19).

### Ethnic Characteristics

A retrospective cohort of large urban centers in the United States studied many HFmrEF patients representing blacks, Hispanics, and whites. From 2008 to 2012, cases of adult patients with HFmrEF were collected from Montefiore Medical Center in the Bronx, New York based on hospitalization echocardiography showing LVEF between 40 and 49%. A total of 1,852 HFmrEF patients (56% male with an average age of 67 years) participated in the study, including 493 non-Hispanic whites (26.6%), 541 non-Hispanic Blacks (29.2%), 489 Hispanics (26.4%), and 329 participants from other ethnic groups (17.8%). Of these groups, white patients tend to be older and less likely to take guide drugs. Compared with the rest of the population, the prevalence of myocardial infarction is lower in Black people. After adjusting for age, gender, and comorbidities, Hispanic individuals had more chronic diseases, but also higher survival rates, than whites and Blacks. There were also significant differences in clinical characteristics between different races/ethnic groups in the HFmrEF group. Non-Hispanic whites with HFmrEF had the highest prevalence of atrial fibrillation. The incidence rate of atrial fibrillation in non-Hispanic whites has been found to be higher than that of non-Hispanic Blacks, Asians, and Hispanics, but the reason for this difference is unclear. As reported, the presence of a large left atrium is associated with a higher prevalence of atrial fibrillation. Many studies have shown that Blacks are more likely to develop coronary heart disease. Compared with whites and Hispanics, Blacks have the lowest levels of NT-proBNP. According to aggregate results from several large community research registries, plasma NT-proBNP levels of Blacks are significantly lower than those of whites due to genetic variation (20).

## Pathophysiological Characteristics

Studies have shown that HFrEF and HFpEF are two different pathophysiological syndromes. HFrEF is usually characterized by systolic dysfunction, while HFpEF is characterized by diastolic dysfunction; however, they often overlap to varying degrees. The pathogenesis of heart failure involves three pathophysiological changes: abnormal activation of neurohormonal mechanisms (4, 21), the disorder of metabolization-inflammatory pathways (22), and dysregulation of cellular signaling mechanisms (23). Based on randomized clinical trials, the following drugs have been shown to reduce cardiovascular endpoints in patients with HFrEF: neurohormone antagonists, SGLT2 inhibitors, cellular, and cGMP-PKG signaling regulators (24). Drugs used to treat HFpEF by reducing EAT inflammation and improving diastolic function include SGLT2 inhibitors, metformin, GLP-1 receptor agonists, and statins. Drugs that can activate cGMP-PKG signaling pathways in HFpEF therapies include Vericiguat (24) and ARNI (25). Further clinical research on the pathogenesis of heart failure caused by metabolic-inflammatory mechanism disorder is required.

**Figure 1:**
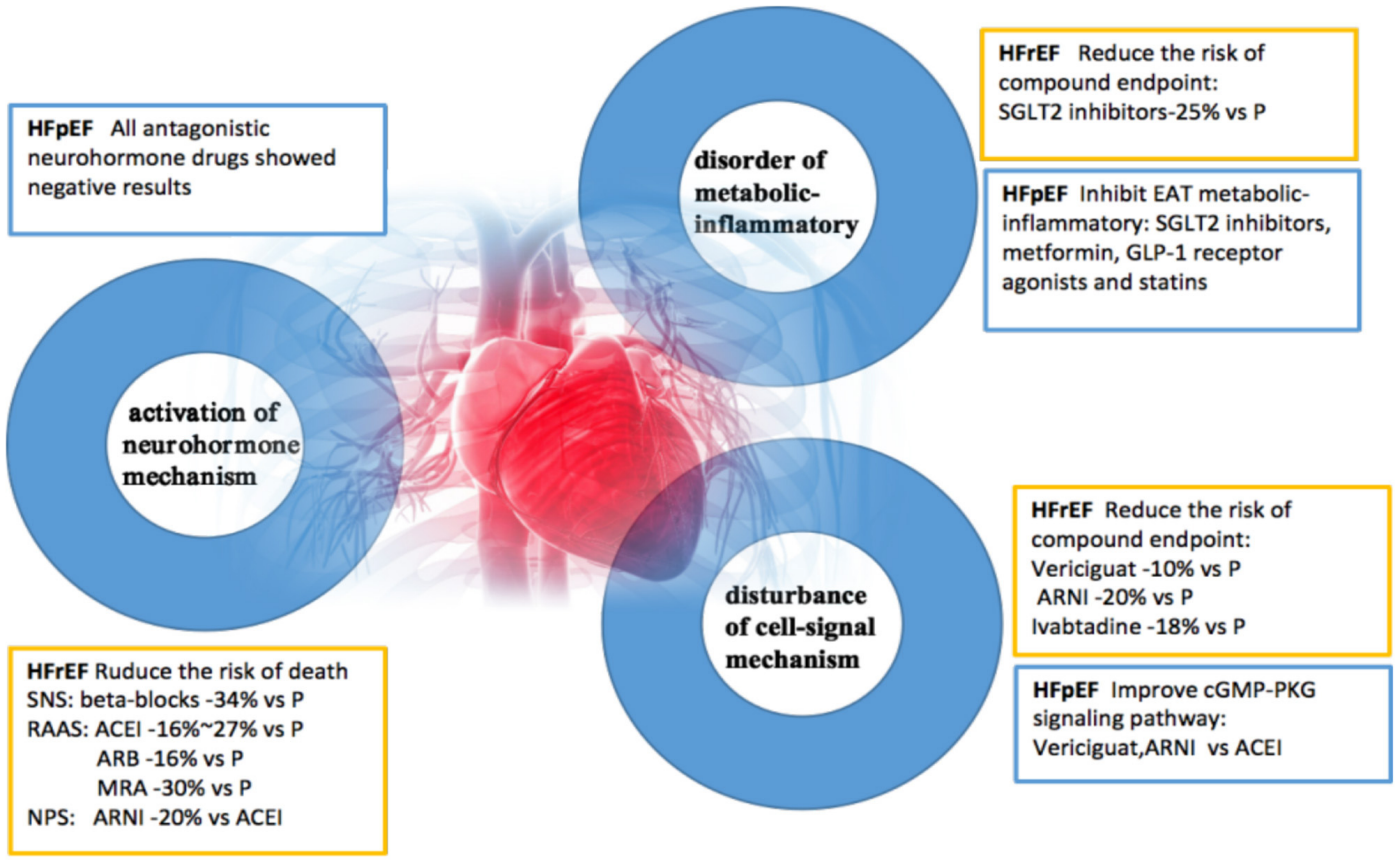
A new understanding of pathogenesis and therapeutic benefits of heart failure. HFpEF, heart failure with preserved ejection fraction; HFrEF, heart failure with reduced ejection fraction; SNS, sympathetic nervous system; RAAS, renin-angiotensin-aldosterone system; NPS, natriuretic peptide system; EAT, epicardial adipose tissue; ACEI, angiotensin-converting enzyme inhibitor; ARB, angiotensin receptor antagonist; MRA, aldosterone receptor antagonist; P, placebo; ARNI, angiotensin receptor enkephalinase inhibitor; SGLT2, sodium-glucose cotransporter 2; GLP-1, glucagon-like peptide-1.

The latest VICTORIA study, presented at the 69th Annual meeting of the American College of Cardiology (ACC2020), adds new evidence to inform drug treatment of high-risk HFrEF patients. Deficiency of cyclic guanosine monophosphate (cGMP) derived from soluble guanylate cyclase (sGC) can lead to myocardial dysfunction and endothelium-dependent vasomotor dysfunction. NO-sGC-cGMP signal pathway has been an important therapeutic target for heart failure. Vericiguat is a novel sGC agonist that directly stimulates sGC production independent of the binding site of nitric oxide, thereby enhancing cGMP level and sensitizing sGC to endogenous nitric oxide (26) (Figure 1).

In fact, in the OPTIMIZE-HF study and other studies, LVEF showed a moderate bimodal distribution in HF inpatients, indicating the presence of two different disease processes (6). As a variable, LVEF shows dynamic change; however, it is by no means arbitrary. Clinical and basic researches suggest that it is appropriate to take such a tangent point of this variable, at least under existing conditions. The TIME-CHF study also showed that left ventricular hypertrophy was caused by concentric remodeling in the HFpEF and HFmrEF groups (albeit to a mild extent), but was caused by eccentric hypertrophy in HFrEF patients (27). The University of Washington Heart Failure Registry compared the degree of diastolic dysfunction between the HFmrEF recovery and deterioration groups, and found the presence of diastolic dysfunction in the deterioration group, indicating that the pathophysiological mechanisms of HFmrEF are heterogeneous (19). In 2016, ESC reported that patients with HFmrEF might present with mild systolic and diastolic dysfunction. The critical question is whether HFmrEF represents a unique clinical entity or just a “transition phase” between HFrEF and HFpEF.

In a study of 110 patients with HFpEF and 61 patients with HFmrEF, two-dimensional speckle tracking echocardiography (2D-STE) was used to evaluate LA phase function. Peak atrial longitudinal strain (PALS), peak atrial systolic strain (PACS), and PAL-PACS were measured to reflect the storage, pumping, and catheter functions of LA, respectively. In patients with normal LA size, LA reserve and pump function were still low for those with HFmrEF. PALS and PACS levels were negatively related to brain natriuretic peptide, LA volume, Emax A, Emax E', systolic blood pressure, and diastolic dysfunction of the pulmonary artery in both groups. Studies have shown that the phase function of LA, as measured by 2D-STE, is worse in patients with HFmrEF than in patients with HFpEF, though the two groups were similar in left atrial size and left ventricular diastolic function as measured by traditional echocardiography (28).

## Clinical Manifestations

### Etiology and Inducement

The Spanish REDINSCORII study shows that the most common risk factor for HFmrEF patients is hypertension, and the most common cause of HF is ischemic heart disease (29). Japanese research groups have suggested that ischemic heart disease is a common cause of HFmrEF. Compared to patients in the HFpEF and HFrEF groups, HFmrEF patients tend to be older, female, anemic, and marked by atrial fibrillation. However, earlier studies suggested that the age, sex, and prevalence of atrial fibrillation and anemia in patients with HFmrEF were intermediate to HFpEF and HFrEF groups.Whereas the prevalence of ischemic etiology was similar to that of HFrEF and higher than that of HFpEF (30).

### Clinical Features

At present, a few results have been reported from trials on HFmrEF patients. These studies only partially include patients with LVEF>45%, while some studies completely exclude patients with LVEF>50%. Nevertheless, insights gained from cohort and enrollment studies help to clarify the clinical characteristics of this group. In 2007, OPTIMIZE-HF studied 41,267 HF patients and analyzed the frequency of hospitalization, demographic characteristics, clinical symptoms, complications, laboratory results, and short-term prognosis based on different LVEF values. This analysis found that patients with LVEF values of 40–50% were more similar to HFpEF patients (6). These results are similar to those of the 2008 ADHERE registration study of patients with LVEF of 40–55%. ADHERE compared the clinical characteristics to those of the other two HF groups and found that the HFmrEF cohort was more similar to the HFpEF cohort in terms of advanced age, female bias, presence of complications [hypertension, COPD, Diabetes Mellitus (DM)], abnormal laboratory indicators (creatinine, B-type natriuretic peptide, troponin) and drug use [beta-receptor blocker, angiotensin-converting enzyme inhibitor (ACEI), angiotensin II receptor antagonist (ARBs)]. However, coronary artery disease was more similar between the HFmrEF group and the HFrEF group. The ADHERE registry also reported different risk factors for these groups. Patients with LVEF>55% are less likely to develop hyperlipidemia, while U.S. patients with atrial fibrillation were more likely to have reduced LVEF. It was also reported that HF patients with LVEF of 40–55% had a higher incidence of myocardial infarction and DM than those with LVEF>55%, and cardiovascular health studies reported that HFmrEF patients had higher rates of diabetes (31).

### Arrythmias

The VIP-HF was an investigator-initiated, prospective, multicentre, observational study of patients with HF and left ventricular ejection fraction (LVEF) >40%. Patients underwent extensive phenotyping, and an implantable loop recorder was implanted later. The primary aim of the VIP-HF study was to examine the incidence of sustained ventricular tachyarrhythmias (VTs) in HF with HFmrEF or HFpEF. Secondary aims were to examine the incidence of non-sustained VTs, bradyarrhythmias, HF hospitalizations, and mortality. It enrolled 113 of the planned 250 patients (mean age 73 yrs, 51% women, New York Heart Association class II/III 54/46%, median NT-proBNP 1,367 pg/ml and mean LVEF 54%; 75% had LVEF >50%). Eighteen percent had non-sustained VTs and 37% had atrial fibrillation on Holter monitoring. During a median follow-up of 657 days, the primary endpoint of sustained VT was observed in one patient. The incidence of the primary endpoint was 0.6 per 100 person-years. The incidence of the secondary endpoint of non-sustained VT was 11.5 per 100 person-years. Five patients developed bradyarrhythmias [3.2 per 100 person-years], three were implanted with a pacemaker. Despite the lower than expected number of included patients, the incidence of sustained VTs in HFmrEF/HFpEF was low. Clinically relevant bradyarrhythmias were more often observed than expected (32).

Although some post myocardial infarction (post-MI) and dilated cardiomyopathy (DCM) patients with HFmrEF face an increased risk for arrhythmic sudden cardiac death (SCD), current guidelines do not recommend an implantable cardiac defibrillator (ICD). They stratified hospitalized HFmrEF patients for SCD with a combined non-invasive risk factors (NIRFs) guiding to programmed ventricular stimulation (PVS) two-step approach. Forty-eight patients underwent a NIRFs screening first-step with electrocardiogram (ECG), Echocardiography and 24-h ambulatory ECG (AECG). Patients were classified as either low risk, moderate risk, and high risk. All in Group 3 received an ICD. After 41 months, 9 of 48 patients, experienced the major arrhythmic event (MAE) endpoint (clinical VT/fibrillation, 3; appropriate ICD activation, 6). The endpoint occurred more frequently in Group 3 than in Group 1 and 2. In hospitalized HFmrEF post-MI and DCM patients, a NIRFs guiding to PVS two-step approach efficiently detected the subgroup at increased risk for MAE (33).

### Echocardiography

All three HF subsets present a similar clinical picture, and the distinction between HFrEF, HFpEF, and HFmrEF ultimately requires an echocardiogram. LVEF is an important index to evaluate the cardiac function of patients with heart failure, and it is closely related to mortality and rehospitalization. However, LVEF is an unstable indicator that may change with treatment and over time. Therefore, LVEF can be regarded as a risk marker of heart failure, but it is by no means the cause of heart failure. The left ventricular cavity and left ventricular myocardial mass gradually increase from HFpEF to HFrEF. In previous studies, LVM was considered as an indicator of cardiovascular events and a prognostic risk factor in HF patients (34). Japanese studies have found that higher LVM may be associated with poor prognosis for patients with HFrEF. TIME-CHF studies showed that left ventricular hypertrophy in HFpEF can be characterized as centripetal hypertrophy, while HFmrEF features mild concentric hypertrophy, and HFrEF features eccentric hypertrophy (27).

In ambiguous cases, a stress test or invasively measured elevated LV filling pressure may be required to confirm the diagnosis. It has been clearer that LVEF may not be the most sensitive parameter to study cardiac function, but may be more accurate to measure myocardial deformation. Although echocardiography is convenient, the measurement of LVEF by echocardiography has inherent issues of variability. Cardiac magnetic resonance imaging is the gold standard for evaluating volume and function (35). Despite these problems, LVEF remains an effective method for HF classification, and previous clinical studies have shown that patients with HFrEF would benefit from the classical treatment of HF compared with the other two HF subgroups (21, 36).

## Treatment and Prognosis

### Drug Treatment

At present, one of the main treatments for patients with HF is a combination of the enkephalin inhibitor sacubitril and valsartan. The PARAGON-HF trial, a global, multicenter, randomized, double-blind, active-controlled trial, included 4,822≥50-year-old HFpEF patients from 43 countries with symptoms and signs of HF, LVEF≥45%, NYHA scores of II-IV in the past 6 months, evidence of structural heart disease, elevated NT-pro-BNP levels and current treatment with diuretics. The purpose of this trial was to investigate the efficacy and safety of ARNI in patients with chronic HFpEF (LVEF≥45%) compared with valsartan. The results showed that, compared with valsartan, ARNI reduced the risk of the primary endpoint by 13%, although it did not reach statistical significance (*P* = 0.059). This study confirmed for the first time clinical benefits existed for some specific subgroups of HFpEF patients, especially those with LVEF<57%. The curative effect also showed population heterogeneity, and the main compound endpoint events in the female subgroup decreased by 27%. In terms of safety, the PARAGON-HF study demonstrated that ARNI is safe and tolerable. The proportion of patients with elevated serum creatinine clearance and enhanced incidence of hyperkalemia were significantly lower than for the control group (37). PIONEER-HF compared ARNI treatment with enalapril treatment in patients with ADHF after hemodynamic stabilization. Compared with the control group, 8-week treatment in the ARNI group significantly reduced the compound risk of severe clinical end events by 46% (HR:0.54, *P* = 0.001), mainly reflected in decreased readmission rate, decreased mortality, and further reduced NT-pro-BNP (38). Therefore, the therapeutic effect of ARNI in HFmrEF patients is promising.

On February 16, 2021, based on data from Phase 3 clinical trials (PARAGON-HF) and Phase 2 clinical trials (PARAMOUNT), as well as on phase 3 HFrEF clinical trial data, FDA formally approved extended indications for chronic heart failure with Sacubitril Valsartan Sodium Tablets (Entresto) to reduce the risk of cardiovascular death and hospitalization in adult patients with chronic heart failure. This decision is a boon for patients with chronic heart failure, resolving the situation that there was no recommended treatment for HFpEF. Both HFrEF and HFpEF patients might benefit from ARNI treatment. The approval of extended indications for ARNI opens a new avenue for the treatment of HFpEF and diastolic heart failure and expands the options for the overall management of chronic heart failure. Why does ARNI expand the indications of chronic heart failure? This drug has two key characteristics. One is that it effectively antagonizes the neurohormone mechanism, the RAS system, and reduces the risk of cardiovascular death and heart failure hospitalization. The other is that it effectively regulates the cardiomyocyte cGMP-PKG signal pathway by protecting natriuretic peptides, improving ventricular diastolic function, and reducing the risk of hospitalization and cardiovascular death from heart failure.

The VICTORIA study explored the efficacy and safety of Vericiguat in high-risk HFrEF patients (24). The results showed that, in addition to the standard treatment of HFrEF, Vericiguat significantly reduces the risk of cardiovascular death or heart failure in high-risk HFrEF patients. Furthermore, Vericiguat is safe and well-tolerated. Administration does not require renal function monitoring or electrolytes and is taken once a day. It was easy to titrate and showed satisfactory drug compliance in the study. As the first sGC agonist, Vericiguat showed positive results in patients with worsening chronic heart failure with decreased ejection fraction, providing a new treatment for patients with HFrEF, and having important theoretical significance and high clinical application value (24).

### Complication Management

**Figure 2:**
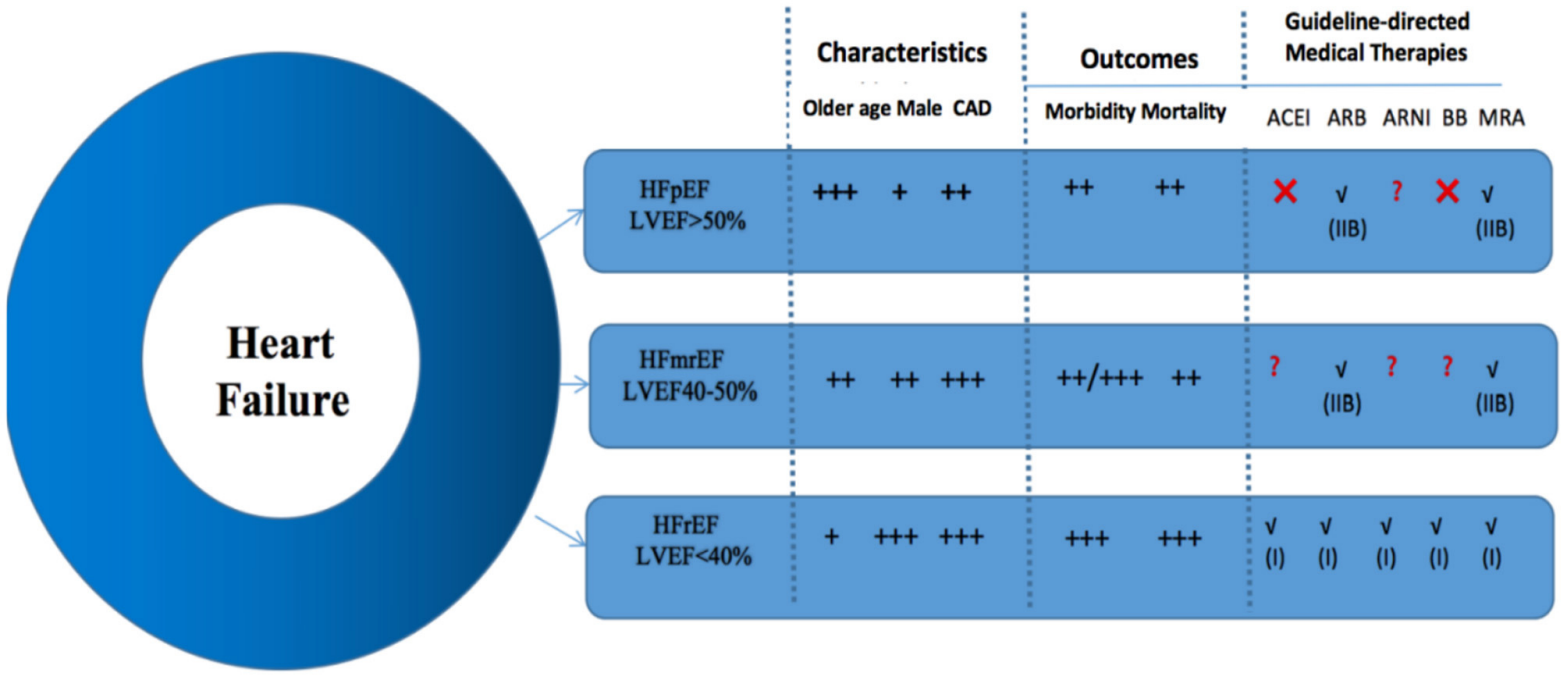
General comparisons of the clinical characteristics, outcomes, and guideline-directed medical therapies for each heart failure group. Class of recommendation is denoted in parentheses, if applicable. ACEI, angiotensin-converting enzyme inhibitors; ARB, angiotensin receptor blocker; ARNI, angiotensin receptor-neprilysin inhibitor; BB, beta-blockers; CAD, coronary artery disease; HFmrEF, heart failure with mid-range ejection fraction; HFpEF, heart failure with preserved ejection fraction; HFrEF, heart failure with reduced ejection fraction; MRA, mineralocorticoid receptor antagonist.

As previously mentioned, the clinical manifestations of complications in HFmrEF patients are more similar to HFpEF patients, and LVEF decrease is more similar to CAD and HFpEF patients (39). Non-cardiogenic comorbidities (COPD, CKD, DM, etc.) are common in HF patients and influence overall incidence. Compared with other groups, uncontrolled hypertension was the most potential cause of readmission in patients with HFmrEF. The usage of ARBs or aldosterone antagonists in patients with HFpEF reduced the readmission rate, possibly by controlling blood pressure and decreasing the risk of LVEF decline in the HFmrEF population (5). Additionally, the DAPA-HF trial confirmed that dapagliflozin, an SGLT-2 (sodium-glucose transporter 2) inhibitor for the treatment of patients with HFrEF, met the preset primary composite endpoint with statistical and clinical significance for a significant reduction in cardiovascular death or worsening of heart failure (40). These drugs provide options for HF patients with DM. From these findings, we boldly infer that SGLT-2 may also be of great significance in improving the symptoms of HFmrEF, especially in delaying the transition from HFmrEF to HFrEF. However, the specific effects require further study (Figure 2).

### Prognosis

At present, research on the prognosis of HFmrEF remains controversial (39). HFmrEF patients may be actually classified as having HFrEF or HFpEF. These distinctions are based on the size and shape of the heart—that is the pathologically morphological characteristics of myocardial remodeling. The main features of HFrEF are cardiac enlargement (especially of the left ventricle), and centrifugal changes in the thinning of the left ventricular wall and interventricular septum. However, the changes of HFpEF usually appeared with normal heart size (or only left atrial enlargement), left ventricular wall, and interventricular septum thickening and concentric hypertrophy conversely. Therefore, if the heart of a patient with HFmrEF is significantly enlarged, this patient may represent an “improved HFrEF”; in other words, the value of LVEF may have increased from <40 to 40–49% after treatment. If the heart size (especially the left ventricle) is normal, this case should be judged as “progressive HFpEF”; that is, the LVEF of the disease has been reduced from ≥50 to 40–49%, indicating that the disease may continue to develop in the future, the heart will expand, and LVEF may be reduced to <40%, making the transition from HFpEF to HFrEF.

Studies of ESC-HF-LT heart failure have found no significant differences in all-cause mortality between HFmrEF, HFrEF and HFpEF (42). The CHART-2 study reported that, for HFmrEF patients, ischemic etiology is related to the decrease of LVEF in the first year, while LVEF is negatively related to death. Therefore, the treatment of CAD may be key to improve the prognosis of patients with HFmrEF (9). According to a Japanese multicenter study, about 1/6 of patients with acute heart failure have HFmrEF (including all-cause death and HF readmission). The combined endpoint and all lethal points were comparable during the 724-day interim follow-up in HFpEF, HFmrEF, and HFrEF patients. Many factors, such as increased age, anemia, hyponatremia, elevated blood urea nitrogen, chronic nephropathy, and increased plasma brain natriuretic peptide levels, have critical prognostic value for HFmrEF patients (43).

The PINNACLE Registry study, a descriptive analysis, found that patients with HFmrEF had more diseases, including coronary and peripheral artery diseases, myocardial infarction, percutaneous coronary intervention, and coronary artery bypass surgery, compared to patients with HFrEF or HFpEF (all *P* < 0.001). Patients with HFmrEF were also more likely to develop chronic kidney disease, diabetes, and atrial fibrillation/flutter. Additionally, these patients generally had a history of smoking (all *P* < 0.001). By LVEF assessment before the analysis, it showed that 4.8% of HFrEF patients converted to HFmrEF, and 32.9% of patients who previously had HFpEF later developed HFmrEF (11). Patients who transition from HFpEF to HFmrEF have a much more complex and less aggressive treatment than those with stable HFmrEF (Table 1).

**Table 1:** Comparisons of clinical characteristics among the different phenotypes of HF.

	Characteristics^*^						**Prognosis**			
	**Age**	**Sex**	**CAD**	**DM**	**HBP**	**AF**	**HOSP****§**	**HOSP-HF**	**DEATH****§**	**CV** **DEATH**
HFpEF	**+++**	**+**	**++**	**+++**	**+++**	**+++**	**?**	**+++**	**++**	**+++**
HFmrEF	**++**	**++**	**+++**	**+++**	**++**	**++**	**?**	**+++**	**++**	**++**
HFrEF	**+**	**+++**	**+++**	**++**	**+**	**+**	**?**	**+++**	**+++**	**++**

*CAD, coronary artery disease; DM, diabetes mellitus; HBP, high blood pressure (hypertension); AF, atrial fibrillation; HOSP, hospitalization; HOSP-HF, hospitalization for HF; DEATH, death from all causes; CV-DEATH, cardiovascular death*;

* *References; (10.21)*,

§ *References (41)*.

## Future Prospects

Further, LVEF may decline over time in patients with HFpEF due to myocardial infarction or inadequate treatment of concurrent cardiovascular disease, and LVEF may be reduced to a lower category. Therefore, HFmrEF is likely to be a heterogeneous category, including patients from different sources and with different clinical characteristics. HFmrEF represents a mixed subcategory that can be divided into HFiEF, HF with stable EF, and HF with deteriorating EF. However, more pathophysiological studies, prospective studies, and retrospective data analysis are needed to further refine these concepts. At present, circulating blood biomarkers and various advanced cardiac imaging models promise to advance research in this field and may guide future treatment options.

Furthermore, due to variability in LVEF measurements based on echocardiography, HF patients may be assigned to the incorrect heart failure groups, confounding the assessed efficacy of treatments in previous studies. Therefore, the LVEF-based classification system and further refinement of specific HF causes (such as ischemia, familial, hypertension) are limited, and detailed phenotypic analysis may help maximize the discovery of more effective treatment strategies. It remains unclear whether the EF classification adopted in the latest version of the guidelines has contributed to further understanding of HF development and improved therapeutic levels.

In the era of precision medicine, the treatment of HFmrEF may include identifying the characteristics of each HF patient, helping to further refine risk stratification beyond individual predictions of LVEF. Advanced imaging models can also identify high-risk patients in the HFmrEF group. Late gadolinium enhancement in CMR could predict death or appropriate (ICD) discharges of implantable cardioverter-defibrillators in patients with heart failure and LVEF>30% (44). A recent study showed that medium-term gadolinium-enhanced CMR is a strong predictor of sudden cardiac death and cardiac arrest (HR35.9) complex endpoints in 40% of patients with dilated cardiomyopathy compared to patients with LVEF of 40–50%, more predictive than LVEF itself (45). Therefore, while studies have demonstrated the potential value of cardiac magnetic resonance imaging evaluation in patients with HFmrEF and HFpEF, further studies are needed to determine whether these late gadolinium-enhanced subgroups can benefit from implanted defibrillators.

In addition, expanding the tools clinicians can use to evaluate hemodynamic variables may improve the prognosis of HFmrEF patients. The application of implantable MEMS pressure sensors in the pulmonary artery can guide the management of patients with heart failure and reduce the rate of hospitalization related to heart failure (46). Additionally, assessment of the biomarkers of patients with heart failure continues to be an active research area.

## Summary

Following the definition of HFmrEF by ACC/AHA and ESC, more studies are needed to explore the mysteries of the “gray area” in HF, including its prevalence, clinical features, and outcomes. Like HFpEF, there are no treatment guidelines for improving the condition of HFmrEF patients. The effective treatment of HFmrEF patients and the special attention to HFmrEF patients may lead to more promising results. The dynamic trend of ejection fraction and other new technology will be provided in the future, thereby confirming whether HFmrEF represents an independent type or a transitional stage between HFrEF and HFpEF.

## Author Contributions

All authors listed have made a substantial, direct and intellectual contribution to the work, and approved it for publication.

## Conflict of Interest

The authors declare that the research was conducted in the absence of any commercial or financial relationships that could be construed as a potential conflict of interest.
